# Quantitative Analysis of SARS-CoV-2 Antibody Levels in Cancer Patients Post Three Doses of Immunization and Prior to Breakthrough COVID-19 Infections

**DOI:** 10.3390/curroncol29100554

**Published:** 2022-09-28

**Authors:** Kathryn Macrae, Jorge Martinez-Cajas, Kristin Bessai, Abulhameed Abdulhamed, Yanping Gong

**Affiliations:** 1 Department of Pathology and Molecular Medicine, Kingston Health Sciences Centre and Queen’s University, Kingston, ON K7L 3N6, Canada; 2 Division of Infectious Diseases, Kingston Health Sciences Centre and Queen’s University, Kingston, ON K7L 3N6, Canada

**Keywords:** SARS-CoV-2, vaccine, oncology, cancer patients

## Abstract

(1) Background: COVID-19 vaccine effectiveness should be carefully evaluated and explicitly defined. To our knowledge, this is the first report to quantitatively evaluate humoral responses post 3 doses of SARS-CoV-2 immunization and prior to breakthrough COVID-19 infection in Canadian cancer patients. (2) Methods: In a prospective cohort study, we enrolled 185 cancer participants post COVID-19 vaccination in Kingston, Ontario, Canada. IgG antibodies against the SARS-CoV-2 spike receptor–binding domain were quantified by immunoassay post three doses of immunization. With the COVID-19 rapid antigen test and polymerase chain reaction (PCR), 16 breakthrough infections were identified. Results: Following SARS-CoV-2 vaccination (including BNT162b2, AZD1222, and mRNA-1273), the mean serum anti-spike protein antibody level was 197.2 BAU/mL (binding antibody unit, SD ± 393.9), 1335.9 BAU/mL (±3337.8), and 3164.8 BAU/mL (±6500.9) post the first, second, and third dose of vaccination. Observed differences were significant (*p* ≤ 0.001). The average antibody level of 3164.8 BAU/mL post the third dose was 89.9 times that of the seroconversion level (35.2 BAU/mL). This indicates that most vaccines approved are effective in producing robust antibody responses. In 11 breakthrough cases confirmed by PCR, prior to infection, the average antibody concentration was 3675.6 BAU/mL with the highest concentration being 9107.4 BAU/mL. Compared with this average antibody concentration of 3675.6 BAU/mL (104.4 times that of the seroconversion concentration), 0% of single dosed, 9.6% of double vaccinated, and 29.5% of triple vaccinated cancer patients had higher SARS-CoV-2 antibody levels. When patients were split into hematological and solid cancer, the hematological cancer group demonstrated lower serological responses than the solid cancer group in the first and second doses (first dose, average concentration 11.1 vs. 201.4 BAU/mL, respectively, *p* < 0.05; second dose, average concentration 441.5 vs. 1725.9 BAU/mL, respectively, *p* < 0.05). There was no difference in the third dose level (1756.3 vs. 2548.0 BAU/mL, *p* = 0.21). (4) Conclusions: Most vaccines were effective in producing robust antibody responses when more than one dose was given, and the more doses the higher the serological response. Likely due to the highly contagious nature of SARS-CoV-2 variants, a significant number of participants had SARS-CoV-2 antibody responses lower than the average antibody concentration prior to the known breakthrough infections. Additional vaccination is likely required to ensure immunity against infection by SARS-CoV-2.

## 1. Introduction

More than two years into the global pandemic of SARS-CoV-2 infection, there are over 12 billion doses of vaccines that have been administered [1]. Currently, Health Canada has approved six vaccines for a national immunization program, e.g., Moderna SpikeVax (mRNA, mRNA-1273), Pfizer-BioNTech Comirnaty (mRNA, BNT162b2), AstraZeneca Vaxzevria (viral vector-based, AZD1222), and Janssen (Johnson & Johnson) (viral-vector based, Ad26.COV2.S) [2]. COVID-19 vaccine effectiveness should be carefully evaluated and explicitly defined in healthy individuals and cancer patients, especially for mRNA vaccines which are based on new technology. 

Cancer patients are at a greater risk of infection compared to healthy individuals. The malignancy and anticancer treatments such as chemotherapy, radiotherapy, or surgery increase their vulnerability to infection. There is a 3.5 folds risk of ICU admission or need for mechanical ventilation for cancer patients compared to patients without cancer [3]. It has been reported that both the clinical outcome and the mortality of COVID-19 in cancer patients are poorer than those in non-cancer [4]. Multiple studies showed that two doses of vaccination showed anti-spike antibody concentrations were significantly lower in cancer patients than in healthy controls [5,6,7]. In addition, there is also a difference in the effectiveness of the vaccine among various cancer patients—a high percentage of cancer patients with solid tumors developed humoral and T-cell responses after vaccinations, whereas patients with hematological malignancies are at higher risk of infection even after the second dose of the COVID-19 vaccine [8].

Based on the extensive knowledge from other vaccination programs, there are multiple markers to evaluate vaccine efficacy. These markers include antibody levels determined by enzyme-linked immunosorbent assay (ELISA), viral and bacterial neutralization assay, interferon assay, and hemagglutination assay [9]. ELISA is the most commonly used methodology to evaluate immunity after immunization [9]. For most other vaccines, a universal cut-off based on semi-quantitative or quantitative ELISA is often chosen to represent protection and immunity [9]. As demonstrated by the Rubella vaccine, the cut-off value should be continuously monitored and adjusted with the aid of large epidemiological studies [10,11]. Due to our limited knowledge regarding the serological responses prior to breakthrough infection, it is unknown if a similar cut-off level for prevention against infection could be selected for SARS-CoV-2 vaccines. 

Limited data exists about serological responses longitudinally post three doses of vaccination, as well as antibody levels prior to breakthrough COVID-19 infections, especially in cancer patients. In this prospective study, we followed immunized cancer patients for antibody responses post three doses and prior to breakthrough infections. To our knowledge, this is the first report to quantitatively evaluate humoral responses post three doses of SARS-CoV-2 immunization and prior to breakthrough COVID-19 infection in Canadian cancer patients. We aimed to determine (1) if additional booster doses further improve their serological responses, (2) if the improved serological responses render protection against infection, (3) if there is a difference in immune responses between solid and hematological cancer, as it is known immunosuppression status is especially pronounced in patients suffering from hematological malignancy since cancer and cancer treatments target immune cells [12]. This knowledge is critical to developing proper public health policies for this vulnerable population.

## 2. Materials and Methods

Institutional ethics committee approval and consent from participants were obtained. In this prospective cohort study from May 2021 to July 2022, 185 cancer patients from the Cancer Centre of Southeastern Ontario were enrolled in the study. A chart review was performed to determine the types of cancer, treatments, and the treatment timeframe. The interval between blood collection and a specific dose was predetermined with the intention of using one single blood collection to represent the likely antibody level before the next dose was offered. Participants were categorized based on the type of vaccine they received for their first, second, and third doses. If a participant received only BNT162b2 for their first, second, and third doses they were placed in the BNT162b2 category. If a participant received only AZD1222 for their first, second, and third doses they were placed in the AZD1222 category. If a participant received only mRNA-1273 for their first, second, and third doses they were placed in the mRNA-1273 category. If a participant received a mixture of BNT162b2, AZD1222, or mRNA-1273 for their first, second, and third doses they were placed in the Mixed Dose category.

The infection status of the study participants was monitored by standard public health protocol in Ontario, Canada. Polymerase chain reaction (PCR) was performed at the Kingston Health Sciences Center microbiology laboratory following standard protocol. Both positive and negative PCR results were charted and reported to the Public Health Ontario database. 

IgG antibodies against the SARS-CoV-2 spike receptor–binding domain were quantified by ELISA (EUROIMMUN, product number: EI 2606-9601-10). The method has been authorized for clinical use by Health Canada and Emergency Use Authorization by the U.S. Food and Drug Administration (FDA). This quantitative method has a linear range between 3.2 to 384 BAU/mL (binding antibody unit). Samples with results over 384 BAU/mL were diluted by a factor of 20 to 30-fold to obtain numeric results. A cut-off of 35.2 BAU/mL was used to determine the seroconversion (recommended by the method manufacturer). 

All statistical analysis was performed using R Statistical Software (the R Foundation, Indianapolis, IN, USA).

## 3. Results

### 3.1. Characteristics of the Study Cohort

The baseline characteristics of study participants are summarized in Table 1. All 185 participants received SARS-CoV-2 vaccines following the recommended dose and dosing interval in Ontario, Canada. The average antibody concentrations were 197.2, 1335.9, and 3164.8 BAU/mL following the first, second, and third dose of vaccination, respectively. On average, there was a 6.8 times increase in antibody concentration from the first to second dose, and 2.4 times increase from the second to third dose. An ANOVA was conducted comparing the average antibody concentration between the three doses, which found a significant difference in these values (*p* < 0.001). 

### 3.2. Characteristics of the Breakthrough Cases

Table 2 describes eight breakthrough cases, representative of all 11 cases identified by PCR. All cases received BNT162b2 for their first, second, and third dose with the exception of case four who received mRNA-1273 for their second dose. The breakthrough infections in relation to dosing and timing of blood collection are detailed in the table. Among infected patients, the highest first dose antibody result was 549.9 BAU/mL, whereas the lowest was 3.2 BAU/mL. It is also interesting to note that the antibody levels varied significantly amongst the second dose antibody results prior to the breakthrough infections. The highest level of antibody generated after the second dose was 5700.9 BAU/mL, whereas the lowest level was 3.2 BAU/mL, with the mean concentration at 2263.3 BAU/mL. Post the third dose, the lowest antibody concentration was 44.8 BAU/mL, while the highest was 9113.1 BAU/mL, with the mean concentration at 4457.0 BAU/mL. This average concentration was 1.4 times the average antibody concentration derived from all participants post the third dose. 

For 16 cancer patients with breakthrough infections (11 identified by PCR, 5 by rapid antigen test), the average antibody concentration prior to infection was 2929.3 BAU/mL, while the highest antibody concentration was 9107.4 BAU/mL. Since it is known that the COVID-19 antigen rapid test has relatively poor clinical sensitivity and specificity when compared with PCR, the further discussion focuses on the eleven breakthrough cases that were confirmed by PCR. Among those 16 cases, fourteen cases occurred between December 2021 to June 2022, when nearly all breakthrough cases were SARS-CoV-2 B.1.1.529 variant (Omicron) in our region, based on phylogenetic analysis of SARS-CoV-2 in Public Health Ontario [13]. 

### 3.3. Antibody Concentration after Different Doses of the Vaccine in Cancer Patients

**Figure 1:**
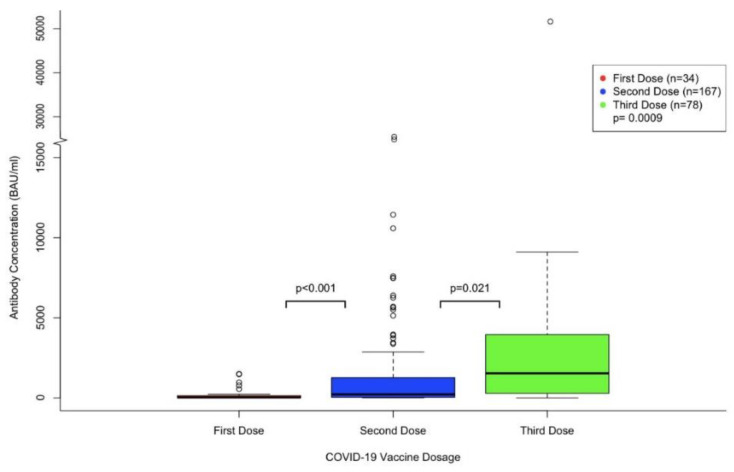
Overall antibody concentration after different doses of the vaccine in cancer patients.

Figure 1 demonstrates antibody levels after receiving a first, second, and third dose with all vaccine types combined. A one-way ANOVA test demonstrated a significant difference amongst the antibody levels when comparing all three doses together (*p* = 0.0009). Additionally, a Welch’s two sample *t*-test was run to compare the first and second dose total antibody levels and demonstrated a significant difference (*p* < 0.001). A Welch’s two sample *t*-test was also run to compare the second and third dose total antibody levels and demonstrated a significant difference amongst the doses (*p* = 0.021).

**Figure 2:**
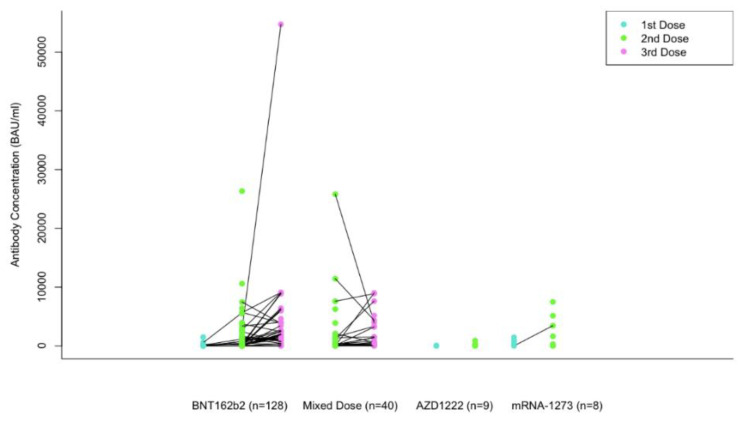
Changes of antibody concentration following first, second and third dose in several types of vaccines in cancer patients.

Figure 2 shows the change in antibody levels after receiving the first, second, and third dose of each participant’s respective vaccines. Each line is drawn from the antibody levels measured after the first dose, to the second, and then the third dose for the same participant. For all vaccine groups, 90.9% of participants demonstrated a higher second dose antibody concentration than the first dose, and 70.8% had a higher third dose antibody concentration compared to the second dose (i.e., 29.2% became lower on the third dose). In the BNT162b2 category, antibody concentration between the first and second dose increased by a factor of 9.3 on average, and 3.4 between the second and third dose. It should be noted that decreases in individual antibody concentration were also observed between the second and third dose. 84.6% of participants who received the BNT162b2 vaccine demonstrated an increase in antibodies between the first and second dose, whereas 77.8% of participants demonstrated an increase in antibodies between the second and third dose. In the Mixed Dose category, antibody concentration increased by a factor of 1.03 on average, between the second and third dose. Additionally, 70.4% of participants demonstrated an increase in antibody concentration, while 29.6% demonstrated a decrease. In the mRNA-1273 category, the average antibody concentration between the first and second dose increased by a factor of 3.2, and 100% of participants who had results recorded for their first and second dose demonstrated an increase in antibody concentration. The cohort of AZD1222 vaccine had limited participants and no comparisons between doses could be made. Factors of increase were not provided in the last two categories due to a limited number of participants. An ANOVA was conducted to compare the first (125.5 BAU/mL), second (1169.3 BAU/mL), and third dose antibody concentrations (3918.9 BAU/mL) of the BNT162b2 group, which demonstrated a significant difference in antibody concentration between each group (*p* < 0.001). A T-test was conducted to compare the second (1839.7 BAU/mL) and third dose (1890.7 BAU/mL) average antibody concentrations from the Mixed Dose category, which demonstrated no significant difference between the antibody concentrations (*p* = 0.96). Due to a limited number of participants, no statistical analyses were performed for the AZD1222 or mRNA-1273 groups.

### 3.4. Vaccine-Specific Serological Responses and Comparison with Antibody Levels Prior to Breakthrough Infections

**Figure 3:**
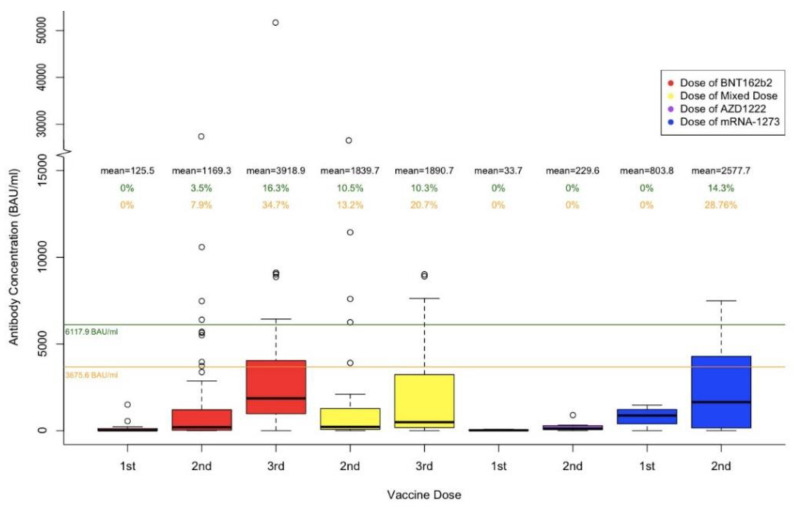
The upper green line of 6117.9 BAU/mL is the second highest antibody concentration prior to a known breakthrough infection (post 3rd dose, blood drawn 39 days prior to PCR confirmed infection). The lower orange line of 3675.6 BAU/mL is the average of antibody concentrations in eleven infections confirmed by PCR. The percentages shown in green represent the percentage of antibody concentration that is above 6117.9 BAU/mL per each dose of each vaccine type. The percentages shown in orange represent the percentage of antibody concentration that is above 3675.6 BAU/mL per each dose of each vaccine type.

Figure 3 shows the mean and the range of participant antibody concentrations after receiving the first, second, and third dose of their respective COVID-19 vaccine. The boxplots are categorized by vaccine types, including BNT162b2, Mixed Dose, AZD1222, and mRNA-1273. To compare the distribution of antibodies in our study population with the antibody levels in breakthrough cases, we arbitrarily chose two potential thresholds (described below). The highest antibody concentration in all breakthrough cases was 9107.4 BAU/mL; however, it was collected 5.7 months (177 days) prior to the infection. We chose to not use this antibody level for comparison as it is known that the antibody levels drop significantly in about six months, up to 25% in one study [14]. When compared with 6117.9 BAU/mL (second highest antibody concentration, blood drawn 39 days prior to PCR confirmed infection and 77 days post third dose), 3.5%, 10.5%, and 14.3% of participants who received BNT162b2, mixed vaccines, and mRNA-1273, respectively, had antibody levels above 6117.9 BAU/mL post the second dose of vaccination. No participants that received the AZD1222 vaccine had antibody levels above 6117.9 BAU/mL. Following a third dose of vaccination, 16.3% and 10.3% of participants who received all doses of BNT162b2 and all mixed doses, respectively, had antibody levels above 6117.9 BAU/mL. When all vaccine groups were combined, the percentage over 6117.9 BAU/mL was 0%, 5.4%, and 14.1% for the first, second, and third dose, respectively.

When compared with 3675.6 BAU/mL (the average antibody concentrations in 11 infections confirmed by PCR), 7.9%, 13.2%, and 28.6%, of participants who received two doses of BNT162b2, two doses of mixed types, and two doses of mRNA-1273, respectively, had antibody levels above 3675.6 BAU/mL. Post the third dose of vaccination, 34.7% and 20.7% of participants who received all BNT162b2 and all mixed doses, respectively, had antibody levels above 3675.6 BAU/mL. When all vaccine groups were combined, the percentage over 3675.6 BAU/mL was 0%, 9.6%, and 29.5% for the first, second, and third dose, respectively.

A one-way ANOVA test demonstrated a significant difference in antibody concentration levels amongst the different vaccine types for the first dose (*p* < 0.002). However, no significant difference was seen in antibody concentration levels amongst the different vaccine types for the second and third dose (second dose, *p* = 0.39; third dose, *p* = 0.18).

### 3.5. Comparison of Hematological and Solid Cancer Types on Antibody Production

**Figure 4:**
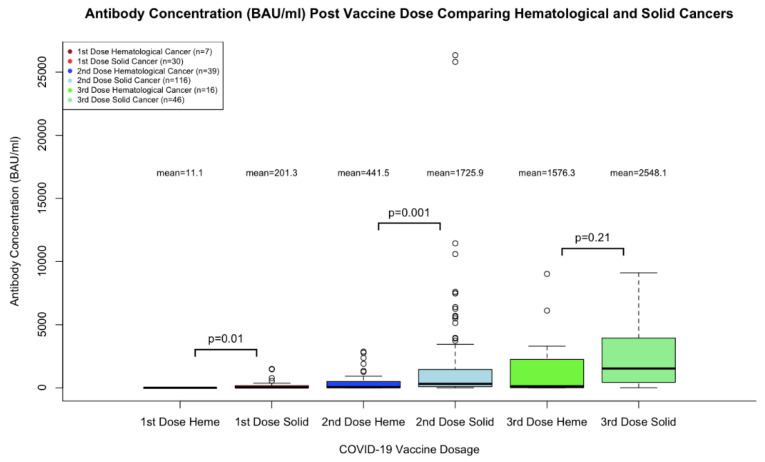
Comparison of Hematological and Solid Cancer Types on Antibody Production.

Figure 4 demonstrates the difference in antibody levels comparing those with hematological and solid cancer types for each dose of the SARS- CoV-2 vaccine. The mean antibody level in BAU/mL is shown for each category. A Welch’s two sample *t*-test was run to compare the antibody levels between those with solid and hematological cancers for each vaccine dose. A significant difference was found when comparing the first dose antibody levels between solid and hematological cancers (11.1 vs. 201.4 BAU/mL, respectively, *p* = 0.01). A significant difference was found when comparing the second dose antibody levels between solid and hematological cancers (441.5 vs. 1725.9 BAU/mL, *p* = 0.001). There was not a significant difference found when comparing the third dose antibody levels between solid and hematological cancers (*p* = 0.21).

## 4. Discussion

Our data demonstrates that when additional dosing of SARS-CoV-2 vaccines were administered in cancer patients, the average antibody levels (for all vaccines combined) continuously rise from the first, second, to a booster dose (*p* < 0.001). This finding concurs with other observations in the healthy population, where similar increases were found [15,16,17,18]. When we categorized the antibody concentration into four groups (BNT162b2, Mixed Dose, AZD1222, and mRNA-1273), we observed a significant statistical difference in antibody concentration in the first dose (*p* < 0.002), but not in the second and third dose (second dose, *p* = 0.39; third dose, *p* = 0.18). This suggests that the difference in vaccine-specific serological response diminishes after multiple doses are administered. The potential public health implication of this finding is that as most of our population has received multiple SARS-CoV-2 vaccinations, it becomes less important to choose a particular vaccine subsequently to achieve a better immune response. Based on this observation, various vaccines should be made easily available and offered widely in Canada, if those vaccines have similar safety profiles. However, this finding of diminished vaccine-specific immune responses after multiple vaccine doses is in agreement with some observations [17] but contradicts others [15]. As the unit of testing results are reported differently (EIA units in [17], U/mL in [15], and BAU/mL in our study), it is also possible that the discordancy in observation may be due to different methods used to measure the antibody levels, that is, the absolute values used in the analysis may have an impact on statistical significance. Standardized and comparable serological testing is essential to evaluate humoral immunity post vaccination. We suggest all methods should be traceable to the WHO International Standard for anti-SARS-CoV-2 immunoglobulin (NIBSC code 20/136) as is our method [19].

In our cohort, there was a 6.8-fold increase in antibody levels from the first to the second dose, and a 2.4-fold increase from the second to third dose. Interestingly, although on average there was a 2.4 times increase in antibody levels from the second dose to the third, 29.2% of our participants demonstrated a lower third dose level when compared with the second. When serological responses are closely monitored at multiple shorter intervals longitudinally, it is known that the antibody levels peak at about four to six weeks, then gradually taper down over time [14,20]. In our cohort, the average interval between the third dose and blood collection was 3.5 months, while it was 1.8 months between blood collection and the second dose. This prolonged interval following the third dose was preselected intentionally to evaluate long-term immunity prior to the next booster dose with a single blood collection. Likely due to this prolonged interval of 3.5 months, some patients demonstrated the third dose antibody levels lower than that of their second dose. Our finding of lower antibody levels at approximately 3.5 months post a third dose in some cancer patients (when compared with the second dose) could inform the public health policy regarding the optimal vaccination interval. In Canada, for individuals at increased risk of severe illness for whom boosters are offered, a shorter interval of at least three months is recommended (compared with an interval of ≥six months for healthy individuals) [21]. The FDA suggests that a second booster may be administered to individuals 50 years of age and older at least four months after receiving a first booster dose [22]. While such recommended intervals are appropriate for the majority of the population at risk, a small percentage of the population may benefit from shorter vaccination intervals to ensure that their antibody levels do not drop significantly. This shorter vaccination interval is also supported by our finding of diminished vaccine-specific immune responses after multiple vaccine doses, which suggests that we should be less selective in the type of vaccine we receive. Clearly, there are multiple factors to be considered in the development of public health policy, and maintaining a high antibody level is only one of those factors.

Another less likely explanation for the decreased third dose serological responses in some cancer patients is that they have reached the peak antibody concentration possible. Their lower antibody levels could be due to analytical variation in the serological method, i.e., their true antibody levels have peaked and only fluctuate slightly between the second and third doses. This hypothesis is supported by data which showed the third dose improved the humoral response in 75% of cancer patients (36 in total) [23], but contradicts findings which showed antibody responses continuously rise from the third to fourth dose in the healthy population [24]. Nevertheless, it is important to understand whether booster doses further increase serological responses or mostly only maintain existing antibody levels in cancer patients. We are currently following up with our participants for the fourth dose antibody measurement.

Adaptive immunity includes humoral immunity, which protects against extracellular microbes and their toxins, and cell-mediated (or cellular) immunity, which is responsible for defense against intracellular microbes. Post-vaccination, it is known that in the absence of antibodies, CD8 + T lymphocytes specific to conserved viral epitopes correlated with cross-protection against symptomatic influenza [25]. A similar phenomenon is also seen in the case of rubella, where low antibody levels may not always be indicative of susceptibility to infection [26]. T lymphocytes comprise a major part of the adaptive immune response to the SARS-CoV-2 virus [27]. Understanding the T lymphocyte response to SARS-CoV-2 can increase our knowledge about the immunogenicity of the vaccines. The assessment of cellular responses relies on time-consuming, laborious, and expensive assays, and as such, are not routinely used. Therefore, serological testing is the primary tool to evaluate the efficacy of most vaccines.

For most other vaccines, a universal cut-off based on semi-quantitative or quantitative ELISA is often chosen to represent protection and immunity. This cut-off is in the range of 1 to 64 times that of the seroconversion concentration [9]. To date, no vaccine developed for other pathogens requires a serological response of more than 64 times the seroconversion concentration to render immunity. The method manufacturer, Euroimmun, recommends a cutoff of 35.2 BAU/mL to indicate seroconversion (confirmed by our unpublished data). The average antibody level of 1335.9 BAU/mL post second dose is 37.9 times that of the seroconversion level, while the average antibody level of 3164.8 BAU/mL post third dose is 89.9 times that of the seroconversion level. This indicates that most vaccines approved are effective in producing robust antibody responses, even in cancer patients who are often immunocompromised due to chemotherapy, radiotherapy, or their medical conditions. Among 185 participants, 16 (8.6%) developed breakthrough infections, which were identified by rapid antigen test and PCR. Among those, eleven breakthrough cases were confirmed by PCR, and the average antibody concentration prior to infection was 3675.6 BAU/mL, while the second highest was 6117.9 BAU/mL, representing 104.4 and 173.8 times that of the seroconversion level, respectively. This suggests that the SARS-CoV-2 virus (especially the Omicron variant) is more contagious than most other pathogens for which we have developed effective vaccines, likely due to their capacities to evade neutralization more efficiently [28].

Before the surge of various variants of concern, SARS-CoV-2 vaccine breakthrough infections occurred in only a small fraction of all vaccinated persons and accounted for a small percentage of all COVID-19 cases [29,30,31]. Prevention against the Delta variant infection was reported at approximately 70% in recent literature [32,33]. Based on recent surveillance data, The Center for Disease Control and Prevention (CDC) has reported symptomatic infection at 37.2% for Omicron post three Janssen mRNA doses at two to four months since the last dose [34]. Breakthrough is observed with other vaccines, such as the influenza vaccine [35]. Many factors likely contribute to the prevention of breakthrough infections, such as adaptive immunity in the host and public health measures. Our data, based on a small cohort, suggests that vaccine mediated antibody response is not the only factor contributing to the prevention of infection, as 91.4% of double vaccinated and 70.5% of triple vaccinated patients had SARS-CoV-2 antibody responses lower than the average antibody concentration of known breakthrough cases. Therefore, effective public health measures (e.g., social distancing or masking being more strictly implemented in cancer patients) likely contributed to our observed lower infected rate at 8.6% (compared with 37.2% from CDC data) in our cancer patients during a 14-month follow-up. Conceivably, a lower serological response is potentially protective against infection when the dose of viral exposure is low through effective public measures [36]. Our findings suggest that different from other vaccination programs, a universal cut-off based on serological response likely is not appropriate for SARS-CoV-2 vaccines as public health measures could further improve immunity to infection in individuals with low serological responses. A larger cohort is required to compare with our findings, which were based on limited participants and breakthrough infections, and therefore are not conclusive. While we acknowledge that the antibody responses in some individuals may not be sufficient to provide protection against infection, the critical role of vaccination in this pandemic could not be underestimated. To highlight this effect, the assessment of vaccine effectiveness should also focus on severe outcomes including hospitalization, ICU admission, or death, and not only breakthrough infections [37,38].

## 5. Limitations

First, the sample size, the heterogeneity of cancer types, and various treatments, did not allow for the comparison based on the cancer type and various treatments. Second, the antibody trend cannot be monitored with a single blood collection post each dose; however, this is the approach (using single serological testing) to evaluate efficacy in other vaccination programs. In this manuscript, we did not test neutralizing antibody concentration. Neutralizing antibodies might represent the best method to evaluate humoral immunity, but their use for routine population-based testing is unpractical due to technical requirements [39], and they do not provide equal protection against all variants [40]. The focus on humoral immunity may not reflect long term immunity in the form of memory B cells or in the T-cell response. Studies to assess memory B cell function and T-cell immunity using assays are underway.

## 6. Conclusions

In cancer patients, most vaccines are effective in producing robust antibody responses when more than one dose is given, and the more doses the higher the serological responses. Likely due to the highly contagious nature of SARS-CoV-2 variants, only 29.5% of triple vaccinated cancer patients had SARS-CoV-2 antibody responses higher than the average antibody concentration prior to known breakthrough cases. The lower antibody levels in many cancer patients even after three doses suggest additional vaccination is likely required to ensure immunity in this vulnerable population.

## Figures and Tables

**Table 1:** Characteristics of the study cohort.

Characteristic	No. (%)	Antibody Concentration, BAU/mL, Mean (SD)
Age, median (age range)	68 (26–93)	
Sex (*n*)		
Male (%)	68 (37.9)	
Female (%)	117 (62.1)	
Solid Tumors	109 (48.9)	
Gastrointestinal	27 (13.2)	
Breast	25 (11.1)	
Genitourinary	17 (6.3)	
Gynecologic	12 (5.8)	
Lung	10 (5.3)	
Melanoma	8 (4.2)	
Head and Neck	5 (2.6)	
Other	5 (2.6)	
Hematologic Malignancy	61 (24.7)	
Lymphoma	22 (11.6)	
Leukemia	17 (5.8)	
Multiple Myeloma	15 (2.6)	
Other	7 (4.7)	
Vaccine Received (n)		
**FIRST DOSE**		
BNT162b2	27	125.5 (298.5)
AZD1222	3	33.65 (38.1)
mRNA-1273	4	803.8 (607.8)
Total and mean	34	197.2 (393.9)
Days between 1st dose and blood collection, mean (SD)	48.2 (±25.8)	
Days between 1st and 2nd dose, mean (SD)	50.9 (±23.2)	
**SECOND DOSE**		
BNT162b2	114	1169.3 (2924.2)
Mixed	38	1839.7 (4645.6)
AZD1222	8	229.6 (290.1)
mRNA-1273	7	2577.7 (2906.0)
Total and mean	167	1335.9 (3337.8)
Days between 2nd dose and blood collection, mean (SD)	55.4 (±33.8)	
Days between 2nd and 3rd dose, mean (SD)	183.1 (±80.0)	
**THIRD DOSE**		
BNT162b2	49	3918.9 (7869.2)
Mixed	29	1890.7 (2718.1)
AZD1222	0	NA
mRNA-1273	0	NA
Total and mean	78	3164.8 (6500.9)
Days between 3rd dose and blood collection, mean (SD)	106.48 (±50.4)	
*p* value	<0.001	

**Table 2:** Characteristics of eight representative breakthrough cases confirmed by PCR.

Age	69	58	58	56	57	70	49	81
Sex	Female	Male	Female	Female	Female	Female	Female	Female
Cancer Type	Breast	Pulmonary	Pulmonary	Colorectal	Breast	Breast	Polycythemia	Cholangio
**FIRST DOSE**								
Days between blood collection and 1st dose (days)	N/A	N/A	57	57	N/A	N/A	N/A	N/A
Antibody (BAU/mL)	N/A	N/A	549.9	3.2	N/A	N/A	N/A	N/A
Interval between 1st and 2nd dose (days)	33	42	80	61	54	N/A	25	24
**SECOND DOSE**								
Interval between blood collection and 2nd dose (days)	15	41	61	68	47	34	22	70
Antibody (BAU/mL)	3.2	1365.3	5640.3	1492.2	5700.9	2754.9	139.2	1010.7
Interval between 2nd and 3rd dose (days)	N/A	213	87	173	210	208	119	205
**THIRD DOSE**								
Interval between blood collection and 3rd dose (days)	N/A	160	17	96	165	163	77	145
3rd dose Antibody Result (BAU/mL)	44.8	1339.8	9107.4	3344.7	3921.3	9113.1	6117.9	2667.0
Interval between blood collection and infection (days)	49	289	177	2	296	186	39	229

The numeric values with underline are used to calculate the average antibody concentration prior to infections, along with three other cases (not shown). The interval between antibody measurement/blood collection and infection is shown on the last row. The antibody concentrations prior to the infection could be either post the second or third dose, as demonstrated in the table.

## Data Availability

The data presented in this study are available on request from the corresponding author.
